# Editorial: Exploring Immune Variability in Susceptibility to Tuberculosis Infection in Humans

**DOI:** 10.3389/fimmu.2021.830920

**Published:** 2022-01-07

**Authors:** Chetan Seshadri, Jayne S. Sutherland, Cecilia S. Lindestam Arlehamn, Julie G. Burel

**Affiliations:** ^1^ Department of Medicine, University of Washington School of Medicine, Seattle, WA, United States; ^2^ Tuberculosis Research and Training Center, University of Washington, Seattle, WA, United States; ^3^ Vaccines & Immunity Theme, Medical Research Council (MRC) Unit, The Gambia at the London School of Hygiene and Tropical Medicine, Banjul, Gambia; ^4^ Center for Infectious Disease and Vaccine Research, La Jolla Institute for Immunology, La Jolla, CA, United States

**Keywords:** *Mycobacterium tuberculosis*, infection, resistance, T cells, biomarkers, co-morbid illness, human immunity

## Introduction

Tuberculosis (TB) occurs along a clinical and immunologic spectrum ranging from exposure and immune sensitization to the highly transmissible pulmonary form of the disease (1, 2). While active disease requires microbiologic confirmation, all other states derive from the use of immunodiagnostics to infer the presence or absence of a paucibacillary infection. The tuberculin skin test (TST) and interferon gamma release assays (IGRA) quantify T cells specific for *Mycobacterium tuberculosis* (M.tb) in the blood, and have been used to define ‘resisters,’ ‘reverters,’ and ‘latent’ infection in humans (3, 4). There is no gold standard for the diagnosis of clinically silent infection with M.tb, and there is limited correlation between immune reactivity and the presence of viable bacilli in humans and animal models (5, 6).

It is against this backdrop that we present the following Research Topic of eleven articles exploring immune variability in susceptibility to tuberculosis infection in humans. The Research Topic spans several themes, including the clinical spectrum of M.tb infection, T cell immunity and transcriptional biomarkers, and the influence of co-morbid illnesses. Together, they advance how we conceptualize and study immunophenotypes of M.tb infection, such as ‘resisters,’ ‘reverters,’ and even ‘latency’.

## Exploring the Spectrum of M.tb Infection in Humans

Two review articles address our evolving understanding of the clinical spectrum of tuberculosis in humans. Guttierez et al. comprehensively review the varying definitions of ‘resisters’ through several household contact and community studies. Based on the heterogeneity in study designs, they argue that a single definition of ‘resistance’ may be inadequate and provide recommendations on how future studies might pursue a more standardized approach. Dubé et al. review the evidence linking human and murine genetic variation in pattern recognition receptors with both the immune response as well as ‘resistance’ to M.tb infection. While genetic variation in this class of genes is clearly associated with altered immunity, it is less clear whether it is associated with hard clinical definitions such as ‘latency,’ which mask the underlying phenotypic heterogeneity of TB. Instead, the authors argue that future studies should be designed to account for heterogeneity by looking for associations with endophenotypes.

## Characterizing M.tb-Specific Immunity

Using longitudinally collected samples, Mpande et al. identified a group of IGRA reverters and compared M.tb-specific CD4 T cell responses with IGRA converters or non-converters from the same cohort. Among IGRA reverters, M.tb-specific CD4 T cells showed a similar activation phenotype but presented an early differentiated phenotype compared to IGRA persistent individuals and more closely resembled phenotypes seen among non-converters. The authors conclude that the magnitude and differentiation status of M.tb-specific Th1 cells among some IGRA reverters may be consistent with well-controlled M.tb infection.

Sharan et al. performed a study of early (one week and three weeks) Mtb-specific T cell responses in the lungs of rhesus macaques after aerosol infection with either a low- or a high-dose of M.tb. The key finding was higher Mtb-specific CD4+IFN-γ+ and TNF-ɑ+ T cell responses in the bronchoalveolar lavage (BAL) after one week in the low dose group that was delayed to three weeks in the high dose group. These results provide insight into T cell priming events occurring very early after infection that depend on bacterial load.

Meier et al. performed a longitudinal assessment of M.tb-specific T cells from HIV-infected individuals up to the time of TB diagnosis. They found that Rv2031c-induced TNF-α was significantly higher in cases compared to controls up to two years prior to TB diagnosis. At time-points closer to diagnosis, they found increased Rv2431 induced IP10 and Rv2031 induced TNF-ɑ in progressors compared to non-progressors. The data provide T cell correlates of risk that may complement blood transcriptional signatures that have been the focus of recent work (7).

Silva et al. investigated cytokine production by peripheral blood immune cells derived from subjects with active, ‘latent,’ or no infection with M.tb after *in vitro* stimulation with M.tb-derived glycolipids. Compared to stimulation with purified protein derivative (PPD), the authors found that glycolipid stimulation elicited diversity beyond canonical Th1 responses to include B cells and CD33+ cells producing several pro and anti-inflammatory cytokines. Further, the magnitude of the cytokine response to M.tb glycolipids was reduced in ‘latent’ or active infection compared to controls suggesting potential hyporesponsiveness upon M.tb exposure, or a mechanism of trained immunity (8).

## Transcriptional Signatures and Biomarkers

Three studies focused on blood transcriptional signatures and other soluble mediators associated with M.tb infection. Baguma et al. assessed a cohort of pre-adolescent children who are intrinsically at lower risk of developing TB disease than post-pubescent adolescents and young adults. They found pre-adolescent children had lower levels of myeloid-associated pro-inflammatory mediators than young adults *in vivo* and after *in vitro* M.tb infection. Wilkinson et al. performed a longitudinal analysis of whole blood and plasma derived from HIV-1 infected individuals during the first six months of antiretroviral therapy. They found a consistent decrease in immune activation and inflammation over this period that may contribute to the reduced incidence of TB after ART initiation. Domaszewska et al. performed a meta-analysis of published whole blood transcriptomes from humans and identified IFN-rich and IFN-low endotypes of TB that did not appear to correlate with the time after M.tb infection in cynomolgus macaques but could distinguish severity of disease.

## Co-Morbid Illnesses

In addition to the studies of HIV co-infection noted above, two studies addressed the role of comorbid illnesses and Mtb infection. Petruccioli et al. examined subjects with immune-mediated inflammatory diseases, who have a high probability of developing active TB, and compared them to individuals with active TB and Mtb infection. They found that among patients with inflammatory diseases, anti-TB therapy did not affect the phenotypes or functions of M.tb-specific CD4 T cells. Diabetes is a major risk factor for progression to active TB and poly (ADP-ribose) polymerase (PARP) activation is mechanistically associated with the development of Type I diabetes. van Doorn et al. used an *in vitro* human macrophage model to investigate the role of PARP inhibitors as host-directed therapy for TB. They find that PARP inhibition decreased survival of both drug-sensitive and drug-resistant M.tb.

## Summary

The work presented in this Research Topic cover the spectrum of diversity of M.tb infection across a variety of human cohorts and illustrate just how far we still need to travel in order to unravel the immunology underlying this diversity. Operational definitions such as ‘resistant’ and ‘latent’ will hopefully give way to molecularly defined endophenotypes that will facilitate better treatments for this global scourge.

## Author Contributions

CS wrote the initial draft. All authors reviewed and edited the manuscript.

## Funding

National Institutes of Health R01-AI146072 to CS.

## Conflict of Interest

The authors declare that the research was conducted in the absence of any commercial or financial relationships that could be construed as a potential conflict of interest.

## Publisher’s Note

All claims expressed in this article are solely those of the authors and do not necessarily represent those of their affiliated organizations, or those of the publisher, the editors and the reviewers. Any product that may be evaluated in this article, or claim that may be made by its manufacturer, is not guaranteed or endorsed by the publisher.
